# Comparison of 3D scanning versus traditional methods of capturing foot and ankle morphology for the fabrication of orthoses: a systematic review

**DOI:** 10.1186/s13047-020-00442-8

**Published:** 2021-01-07

**Authors:** Muhannad Farhan, Joyce Zhanzi Wang, Paula Bray, Joshua Burns, Tegan L. Cheng

**Affiliations:** 1 Engineering Prototypes & Implants for Children (EPIC) Lab, The Children’s Hospital at Westmead, Sydney, NSW Australia; 2 University of Sydney School of Health Sciences & Children’s Hospital at Westmead, Westmead, Sydney, NSW 2145 Australia; 3 Faculty of Medical Rehabilitation Science, Taibah University, Al Madinah Al Munawarah, Saudi Arabia; 4 The University of Sydney Children’s Hospital Westmead Clinical School, Faculty of Medicine and Health, The University of Sydney, Sydney, Australia

**Keywords:** 3D scanning, Lower limb, Lower extremity, Impression, Orthoses, Orthotic devices

## Abstract

**Background:**

In the production of ankle-foot orthoses and in-shoe foot orthoses, lower leg morphology is traditionally captured using a plaster cast or foam impression box. Plaster-based processes are a time-consuming and labour-intensive fabrication method. 3D scanning is a promising alternative, however how these new technologies compare with traditional methods is unclear. The aim of this systematic review was to compare the speed, accuracy and reliability of 3D scanning with traditional methods of capturing foot and ankle morphology for fabricating orthoses.

**Methods:**

PRISMA guidelines were followed and electronic databases were searched to March 2020 using keywords related to 3D scanning technologies and traditional foot and ankle morphology capture methods. Studies of any design from healthy or clinical populations of any age and gender were eligible for inclusion. Studies must have compared 3D scanning to another form of capturing morphology of the foot and/or ankle. Data relating to speed, accuracy and reliability as well as study design, 3D scanner specifications and comparative capture techniques were extracted by two authors (M.F. and Z.W.). Study quality was assessed using the Grading of Recommendations, Assessment, Development and Evaluations (GRADE) and Consensus-Based Standards for the Selection of Health Measurement Instruments (COSMIN).

**Results:**

Six articles met the inclusion criteria, whereby 3D scanning was compared to five traditional methods (plaster cast, foam impression box, ink footprint, digital footprint and clinical assessment). The quality of study outcomes was rated low to moderate (GRADE) and doubtful to adequate (COSMIN). Compared to traditional methods, 3D scanning appeared to be faster than casting (2 to 11 min vs 11 to 16 min). Inter-rater reliability (ICC 0.18–0.99) and intra-rater reliability (ICCs 0.25–0.99) were highly variable for both 3D scanning and traditional techniques, with higher agreement generally dependent on the foot parameter measured.

**Conclusions:**

The quality and quantity of literature comparing the speed, accuracy and reliability of 3D scanning with traditional methods of capturing foot and ankle morphology is low. 3D scanning appears to be faster especially for experienced users, however accuracy and reliability between methods is variable.

**Supplementary Information:**

The online version contains supplementary material available at 10.1186/s13047-020-00442-8.

## Introduction

Orthoses are external supportive devices that are worn to reduce pain and enhance the function of patients with disorders of the neuromuscular and/or musculoskeletal systems. The most common types of orthoses are hand splints, spinal bracing, in-shoe foot orthoses (FO), and ankle-foot orthoses (AFO). FOs are used to support and accommodate the foot to avoid or correct foot deformities, help to maintain uniform body weight distribution and improve foot function as well as reduce pain. AFOs aim to regulate the movement of the ankle, compensate for weakness, heel misalignment and control deformities. AFOs are also used to improve toe clearance, maintain a neutral position and resist contracted muscles of the ankle and foot during gait.

Traditional manufacturing of foot and ankle orthoses is cumbersome and involves labour intensive manual production methods [1]. Fabrication of FOs involves first forming the negative impression, such as pressing the plantar surface of the foot into a foam box or draping the foot using plaster of Paris. The negative impression is filled with plaster to generate the positive model and manually modified by the clinician. After placing the positive model on the vacuum table, it is wrapped with heated thermoplastic sheet and moulded to the geometry of the model by applying a vacuum. Similarly, AFOs are generally hand made from plaster casting the patient’s lower leg. The negative cast is filled with fluid plaster of Paris to form a positive model, which is modified manually by adding or removing plaster. This modified positive is then vacuum formed with polypropylene to produce an AFO [2, 3]. These traditional methods of capturing foot and ankle morphology can be highly resource intensive, especially for fabricating AFOs, requiring dedicated infrastructure including a casting room, plaster friendly furniture, sink with plaster capture, and anti-slip floor. For departments or practices that have only one casting room and several clinicians, room availability can present logistical difficulties and increased patient wait-times. In addition, storage of consumables, such as foam impression boxes and plaster rolls, requires a dry area that can occupy a large space. Positive plaster casts are heavy and need to be handled carefully to prevent breakage that would require recasting the patient. After fabricating either FOs or AFOs, the positive cast is destroyed to provide storage space for new casts, which prevents duplication of the same prescription from an old cast. Although casting for FOs may not require the same level of resourcing as AFOs, the process is still highly dependent on practitioner experience of positioning and manipulating the foot and/or ankle [4]. Overall, these traditional methods are labour intensive, restrict design choices, and require high level of skill and dedicated infrastructure that can increase costs and patient wait times [5, 6].

Digital approaches are emerging as alternative plaster-less fabrication methods, which have the potential to reduce long lead times, material wastage, and cost [5, 7]. Digital fabrication requires the input of foot and ankle morphology, which can be obtained by 3D scanning the foot and ankle directly or by scanning a negative impression. The digitised data can be used to create a 3D model of the patient and/or their device [8]. Computer Aided Design (CAD) software is used to process the 3D scan, where adjustments can be made to the geometry [9]. The resulting 3D model of the modified positive model or final orthotic design can then be exported to either a milling machine or a 3D printer [10, 11].

Recently, 3D scanners have been developed to improve the production of orthoses and prostheses [12, 13]. Using 3D scanning provides clinicians with flexibility as to where they are taking impressions (e.g. on ward or in fitting rooms or in the community). 3D scanning technology would also minimise plaster storage requirements and enable exact duplication of a prescription. In a digital fabrication workflow, the accurate and reliable input of a patient’s lower leg morphology is critical for an appropriately fitting device. However, commercially available scanners have varying speed, accuracy and reliability that need to be investigated. Therefore, the aim of this systematic review was to compare the speed, accuracy and reliability of 3D scanning with traditional methods of capturing foot and ankle morphology for fabricating orthoses.

## Methods and materials

### Systematic literature search

We followed the Preferred Reporting Items for Systematic Reviews and Meta-Analyses (PRISMA) checklist to conduct the systematic review [14]. The search strategy focused on terms comparing 3D scanning with other traditional morphology capture methods of the foot and/or ankle. The focus was to cover all possible methods for capturing the morphology of the foot and ankle. For example, plaster casting and foam impression boxes could be used to capture the foot and ankle to produce AFOs, FOs or footwear.

Search terms related to the 3D scanning (3D scan*, Photogrammetry, Laser scan*, Structured Light scan*, Three dimensional scan*, (3D digitalization or 3D digitalisation), Three-dimensional construction, 3D surface scan*, 3D laser scan*, 3D structured light scan*, scan*) combined with AND to search terms related to foot and/or ankle (ankle, foot, Foot morphology, (ankle and foot), ankle morphology, foot parameter*, ankle parameter*) also combined with AND to search terms related to foot and ankle capturing methods other than 3D scanning (Plaster cast*, foam impression, polyurethane resin, (fiber* cast* or fibre* cast*), (orthopedic cast* or orthopaedic cast*), cast*, (Plaster Mold* or plaster Mould), (mold* or mould), Hand cast*, (cast* adj4 ankle), (cast* adj4 foot), fiberglass cast*, cast* socks, negative impression, positive impression, foot trace*, foot print*, footprint). Electronic database searches were carried out in ProQuest Central, MEDLINE (via OvidSP), EMBASE, Scopus, AMED (via OvidSP), Web of Science and CINAHL (via EBSCO). The search keywords were designed for MEDLINE and adapted for other databases. No restrictions were added to the search, such as language or date (Additional file 1).

### Eligibility criteria

All studies comparing 3D scanning to other foot and/or ankle capture methods were eligible for review and were included regardless of date of article publication, language, participant age or demographics. Reviews, conference reports and newspaper articles were excluded, as these are rarely peer-reviewed and may only present preliminary or limited data.

### Study selection and data extraction

After deletion of duplicates, the titles and abstracts of included studies were assessed and screened by authors M.F. and Z.W. based on eligibility criteria in the first pass. Following this, the full-text articles were retrieved by the researchers to assess eligibility for inclusion independently (second pass). In case of disagreement, author J.B. was asked to evaluate the article and all discrepancies were resolved. The included articles underwent data extraction by authors M.F. and Z.W. independently who then compared the extractions for accuracy and completeness. Extraction of data comprised of study design, sample size, demographic characteristics, information about the 3D scanner and scanning methods, and comparative foot/ankle capture techniques.

Study outcomes of time, accuracy and reliability were extracted. For the purposes of this review, we considered speed as the time required to obtain the foot and/or ankle morphology, accuracy as the relationship between the scanned measurements to the patient’s clinical measurements, and reliability as the consistency and repeatability of the capture method. The reported ICCs were classified as poor (less than 0.50), moderate (between 0.50 to 0.75), good (between 0.75 to 0.90) or excellent (greater than 0.90) [15].

### Study quality

The evidence level of each study was assessed using the Oxford Centre for Evidence-Based Medicine 2011 Levels of Evidence (OCEBM Levels) rating from level one to level five, where level one representing a randomised controlled design and highest level of evidence [16]. The methodological and quality of the assessment measures reported in the included studies were rated using version one of the Consensus-Based Standards for the Selection of Health measurement Instrument (COSMIN) [17–19]. The reported instruments were assessed using three measurement properties from the COSMIN checklist: content validity, reliability and measurement error [20]. These three items from the COSMIN checklist were used as they were the most relevant to the aims of this review. COSMIN is the gold standard for patient reported outcome measurement evaluation, however more recently it has been applied to clinician-rated assessments [17, 20, 21]. Studies that evaluated or reported the efficacy of an intervention were assessed using the Grading of Recommendations Assessment, Development, and Evaluation (GRADE) approach [22] utilising GRADEpro GDT. The GRADE approach was used to assess the strength and certainty of the overall evidence reported in the included studies in this review.

### Meta-analysis

We attempted meta-analysis on pooled measurements of foot parameters collected from 3D scanning and traditional methods. Three studies [23–25] provided raw measurements for parameters of the foot such as arch height, rearfoot width and forefoot width. However, due to heterogeneity in data capture methods we were unable to conduct the meta-analysis (Additional file 2).

## Results

### Description of included studies

**Fig. 1:**
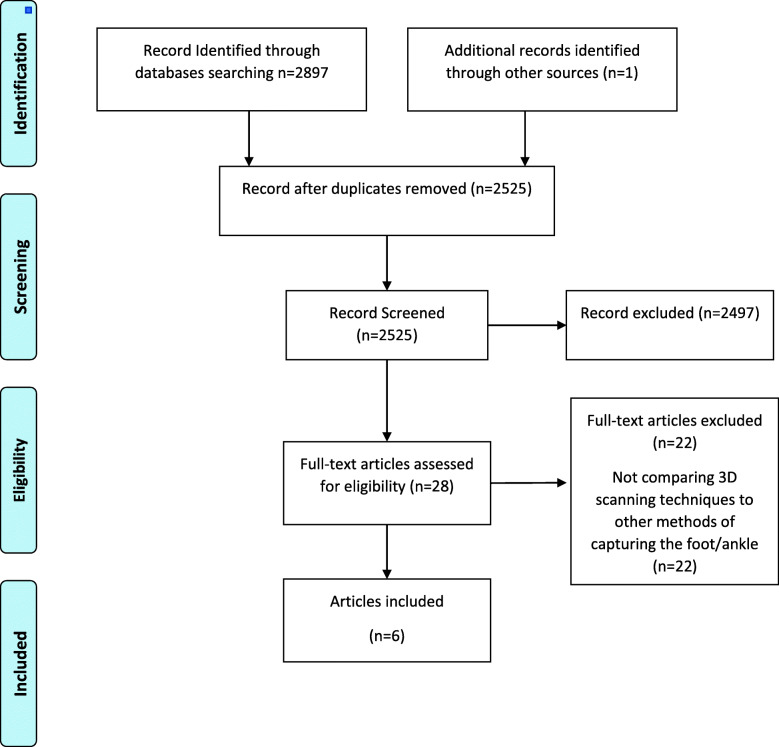
Flow diagram of the search history and selection process

The original electronic database search led to a total of 2897 articles with 2525 publications remaining after eliminating duplicates. After completing the title and abstract evaluation, full-text articles were downloaded for 26 publications. After screening and analysing the article’s full text, only five met the inclusion criteria and were included in this review (Fig. 1). One additional article was found after a hand search of the references. The six studies included one randomised controlled trial evaluating AFOs [26], four studies reporting reliability and accuracy of 3D scanning for FOs [23–25, 27], and two studies assessing speed of 3D scanning for FOs and AFOs [25, 28] (Table 1). Traditional foot and ankle capture tools included in the studies were plaster cast, foam impression, digital footprint, ink footprint and 3D scanning. The 3D scanning technologies found in this systematic review included laser 3D scanners (Table 1).

**Table 1:** Participant characteristics and outcomes of included studies

Reference	Participant characteristics	Orthotic device	Comparison methods and weightbearing status	3D scanner name and technology	3D scan position and weightbearing status	Outcomes	Main result and authors conclusions	OCEBM level
Roberts, et al. 2016 [26]	*N* = 136 aged < 21 yrs., prescribed rigid and/or hinged AFOs (10.7 SD 4.9 yrs. for males, 9.8 SD 4.1 yrs. for females)	AFO	Plaster cast, casting position and weightbearing status not described	3D FastSCAN (Laser scanner)	Position and weightbearing status not described	1) Secondary outcome measures time (min) spent with subjects to cast and scan limbs	1) Orthotists experienced in 3D scanning had a significant time reduction compared to casting	2
Carroll, et al. 2011 [24]	*N* = 21 aged > 20 yrs., healthy participants (35.4 SD 13.6 yrs)	FO	Plaster cast, NWB (sitting)	Name not provided (3D non-contact digitiser)	NWB (sitting)	1) ICC for intra and inter-rater reliability 2) Measurement error assessed by smallest real difference	1) 3D scanning is reliable with reduced measurement variability 2) Smallest real differences consistent between the raters and casting technique	3
Laughton, et al. 2002 [23]	*N* = 15 aged 20–34 yrs., free of lower-extremity injuries (23.8 SD 3.6 yrs)	FO	1) Plaster cast, NWB (lying prone) 2) Foam impression, PWB (sitting)	Sharp Shape (Laser scanner)	1) PWB, (sitting) 2) NWB, (lying prone)	1) Reliability as assessed by within-method ICCs 2) ICCs between clinical measures and the four methods	1) Methods differ in reliability 2) Accuracy of foot measures are influenced by the method used	3
Telfer, et al. 2012 [27]	*N* = 22 aged > 18 yrs., with non-cavus foot type (42.8 SD 11.4 yrs)	FO	1) Plaster cast, NWB (lying prone) 2) Foam box, PWB (sitting) 3) Foam box, FWB (walking into the box)	3D FastSCAN (Laser scanner)	1) 50% WB (relaxed standing position) 2) 50% WB (Corrected standing) 3) PWB (sitting)	1) Intra- and inter-rater reliability (ICC (2,1))	1) Apart from medial arch height, all methods shows good-excellent intra- and inter-rater reliability	3
Lee, et al. 2014 [25]	*N* = 130 age 18–30 yrs., Healthy participants (21.25 SD 2.15 yrs. for males, 21.98 SD 2.94 yrs. for females)	Not described	1) Digital calliper, 50% WB (standing) 2) Ink footprint, 50% WB (standing) 3) Digital footprint, 50%WB (position)	INFOOT USB (Laser scanner)	50% WB (position)	1) The mean absolute difference values 2) ICCs for precision evaluation	1) 3D scanning had lowest mean absolute difference 2) Apart from ink footprint, all measures had ICCs from good to excellent	3
Payne, 2007 [28]	? - No participant characteristic or condition were given	FO	Plaster cast, casting position and weightbearing status not described	Name not provided (Technology type not reported)	Position and weightbearing status not described	1) Time to 3D scan and plaster cast of the foot by experienced podiatrist and student	1) Plaster casting slower than 3D scanning, especially for experienced user	5

*NWB* non-weightbearing, *PWB* partial-weightbearing, *FWB* Full-weightbearing, *50%WB* 50% weightbearing,

### Participant characteristics

The six studies included 189 adults (102 female, 86 male, and 1 unspecified) [23–25, 27, 28] and 134 children (59 girls, 75 boys) [23, 24, 26, 27]. The mean age and SD of the participants in the included studies were 23.8 years, SD 3.6 [23], 35.4 years, SD 13.6 [24], 42.8 years, SD 11.4 [27], males 21.2 years, SD 2.1 and females 21 years SD 5.9 [25], males 10.7 years SD 4.9 females 9.8 years SD 4.1 [26] and not reported [28]. The sample size for the studies were *n* = 15 [23], *n* = 21 [24], *n* = 22 [27], *n* = 130 [25], *n* = 134 [26] and n = 1 [28]. Five studies included healthy participants [23–25, 27, 28] while only one study included participants who were prescribed with rigid and/or hinged AFOs although their conditions were not reported [26] (Table 1).

### Capture methods

All six studies compared 3D scanning to other methods of capturing foot and/or ankle morphology. Two studies compared 3D scanning with both plaster casting and foam impression [23, 27]. Three studies compared 3D scanning to plaster casting methods only [24, 26, 28]. One study compared 3D scanning measurement to digital and ink footprints as well as digital calliper measurements [25]. The included studies assessed several weightbearing positions including non-weightbearing, full weightbearing, partial weightbearing and 50% weightbearing (Table 1).

### Quality of included studies

The extracted outcomes were assessed using the GRADE process, with overall quality evidence for rearfoot width and medial arch height measurement outcomes considered very low, the forefoot width outcome considered low indicating weak evidence for reliability and accuracy (Table 2). There was a moderate level of quality for scanning and casting time for ankle and foot, showing reasonable evidence for speed. There was a very low level of quality for scanning and casting time for the foot only [28] (Table 1). Four of the studies were classified as OCEBM level 3 [23–25, 27], one was level 2 [26], and one was level 5 [28].

### Quality and measurement properties of included assessments

The quality of three measurement properties included in the four studies [23–25, 27] was rated using the COSMIN checklist, reliability, measurement error and criterion validity. Based on Terwee (21, 22), the COSMIN checklist was rated using quality criteria (Table 3). The quality of outcome and measurement property were rated and shown in Table 4. Methodological quality and outcome quality of each tool was assessed using the COSMIN checklist in several measured outcomes such as forefoot, rearfoot and arch height to assess the accuracy and reliability between methods (Table 5).

**Table 2:** GRADE evidence profile

Certainty assessment							№ of patients		Effect	Certainty	Importance	
№ of studies	Study design	Risk of bias	Inconsistency	Indirectness	Imprecision	Other considerations	3D scanning	traditional methods	Relative (95% CI)	Absolute (95% CI)		
**Forefoot width**												
[23–25, 27]	observational studies	not serious	serious^a,b^	not serious	not serious	none	203	203	–	not estimable	⨁◯◯◯ VERY LOW	IMPORTANT
**Rearfoot width**												
[23–25, 27]	observational studies	not serious	serious^a, **c**^	not serious	serious^b^	none	203	203	–	not estimable	⨁◯◯◯ VERY LOW	IMPORTANT
**Arch height (medial)**												
[23, 24, 27]	observational studies	not serious	serious ^a, **c**^	not serious	serious^b^	none	73	73	–	not estimable	⨁◯◯◯ VERY LOW	IMPORTANT
**Time spent to cast or scan for foot and ankle (min)**												
[26]	randomised trials	not serious	not serious	not serious	serious^b^	none	64	70	–	MD 1.33 min higher (0.4 lower to 3.1 higher)	⨁⨁⨁◯ MODERATE	CRITICAL
**Time spent to cast or scan for foot (min)**												
[28]	observational studies	very serious^d,e^	not serious	not serious	very serious^f^	none	1	1	–	not estimable	⨁◯◯◯ VERY LOW	IMPORTANT

*CI* Confidence interval, *MD* Mean difference

Explanations

^a^Large variation in effect. Some suggest 3D scanning, while some for plaster casting

^b^Wide 95% CI value which includes favours of both 3D scanning and plaster casting

^c^PHigh I-squared value

^d^No description of randomization and lack of blinding

^e^PA very small sample size

^f^Very few events and no reports of CIs

**Table 3:** Quality criteria for study outcomes for COSMIN. Based on Terwee et al. [29]

Property	Rating	Outcome Quality Criteria
Criterion validity	+	Convincing arguments that criterion standard is “gold” AND correlation OR ICC with criterion standard ≥0.70.
	?	No convincing arguments that gold standard is “gold” OR no correlations OR ICC have been calculated.
	–	Correlation OR ICC with criterion standard < 0.70, despite adequate design and method.
Reliability (intra-rater, inter-rater)	+	ICC or weighted Kappa ≥0.70 OR Pearson γ or Spearman ρ ≥ 0.80 with evidence provided that no systematic change has occurred.
	?	No ICC, weighted Kappa, Pearson γ or Spearman ρ is determined OR doubtful design^a^.
	–	ICC or weighted Kappa < 0.70 OR Pearson γ or Spearman ρ < 0.80, despite adequate design and method.
Measurement error	+	SEM < SDC OR SEM outside the LOA
	?	SEM is not defined OR doubtful design^a^.
	–	SEM ≥ SDC OR SEM equal or inside the LOA

*Abbreviation*: *ICC* intraclass correlation coefficient, *SEM* standard error of measurement, *SDC* smallest detectable change, *LOA* limits of agreement

^a^Doubtful design = any essential weakness in design or execution of the study

**Table 4:** Levels of evidence for the quality of the measurement property for COSMIN

Level	Rating^a^	Criteria
Strong	+++ or —	Consistent findings in multiple studies of adequate methodological quality OR in one study of very good methodological quality.
Moderate	++ or –	Consistent findings in multiple studies of doubtful methodological quality OR in one study of adequate methodological quality.
Limited	+ or -	One study of inadequate methodological quality.
Conflicting	+/−	Conflicting findings.
Unknown	?	Only studies of inadequate/doubtful methodological quality.

^a^NOTE. + = positive;? = indeterminate; − = negative [17]

Using the COSMIN checklist, the intra-rater reliability was scored moderate for weightbearing 3D scanning and digital footprint (ICC > 0.70) and the ink footprint rating level was moderate in measuring foot parameters (ICC > 0.70). The other capturing methods rated unknown for the intra-rater reliability as the methodological quality of the included studies where either inadequate or doubtful (Table 5). The measurement error and criterion validity rating level were unknown due to inadequate number of participants *N* < 30 and inadequate or doubtful methodological quality (Table 5 and Additional file 3).

### Speed

Two studies compared the time required to 3D scan versus plaster cast the foot and ankle (Table 6) [26, 28]. In one study, the overall time taken for 3D scanning (11.1 mins, SD 9.5) and casting (12.5 mins, SD 4.9) was similar (*p* = 0.056) [26]. However, the subset of clinicians with experience in 3D scanning were faster at 3D scanning than casting: scanning time (8.9 mins, SD 2.9) and casting (13.1 mins, SD 4.4) (*p* < 0.001) [26]. The second study compared the time for 3D scanning and casting both feet [28], with the bilateral foot scan taking 2 mins compared to casting taking 11 mins for an experienced clinician and 16 mins for a student (Table 6). However, this study did not report the number of participants included, standard deviation or statistical analyses.

**Table 5:** Assessment of the methodological quality of the studies and outcome quality of the measurement property for each method using COSMIN checklist

Method	Reference	Measurement property: methodological quality per study		
		Reliability	Measurement error	Criterion validity
Casting				
NWB	[23, 24, 27]	?	?	?
Foam impression				
PWB	[23, 27]	?	?	?
FWB	[27]	?	?	
Ink footprint				
50% WB	[25]	--^a^	?	?
Digital footprint				
50% WB	[25]	++^a^	?	?
3D Scanning				
NWB	[23, 24, 27]	?	?	
PWB	[23, 27]	?	?	?
PWB (corrected position)	[27]	?	?	
50% WB	[25]	++^a^	?	?

*Abbreviations*: *NWB* non-weightbearing, *PWB* partial-weightbearing, *FWB* Full-weightbearing, *50%WB* 50%. weightbearing

Outcome Quality: + = positive, − = negative,? = unknown

^a^One study had adequate methodological quality

### Accuracy

One study assessed the accuracy of foot measures from 3D scans and traditional methods against clinical measurements, using ICCs [23]. Partial-weightbearing 3D scanning was found to accurately capture forefoot width (ICC 0.93) and rearfoot width (ICC 0.77), while non-weightbearing 3D scanning was accurate for forefoot width (ICC 0.84), however poor for rearfoot width (ICC 0.43). For the traditional methods, non-weightbearing plaster casting was accurate when capturing rearfoot width (ICC 0.77) and forefoot width (ICC 0.88) and partial-weightbearing foam impression was good for forefoot width (ICC 0.88), however poor for rearfoot width (ICC 0.46) [23]. Another study measured the accuracy of 3D scanning, digital footprint and ink footprints against digital caliper measurements by reporting the mean absolute difference values, finding that 3D scanning ranged between 0.6 to 11.9 mm while the two footprint methods ranged from 0.1 to 14.9 mm (no statistical analyses reported) [25].

### Inter-rater reliability

Two studies reported inter-rater reliability of foot length, forefoot width, rearfoot width and medial arch height [24, 27] (Additional file 4). Carroll et al. found non-weightbearing 3D scanning demonstrated good to excellent ICC ratings (ICC_3,1_ 0.81–0.99), while non-weightbearing casting measures of reliability ranged from poor to excellent (ICC_3,1_ 0.57–0.99) [24]. Telfer et al. reported the position of 3D scanning affected inter-rater reliability, with relaxed standing ranging from good to excellent (ICC_2,1_ 0.73–0.92), corrected standing ranging from poor to excellent (ICC_2,1_ 0.35–0.94) and corrected sitting ranging from good to excellent (ICC_2,1_ 0.75–0.92). In the same study, inter-rater reliability of traditional methods also varied with position, with plaster casting ranging from poor to moderate (ICC_2,1_ 0.64–0.89), foam impression when sitting and walking ranging from poor to excellent (ICC_2,1_ 0.41–0.91) [27].

### Intra-rater reliability

Four papers reported intra-rater reliability measures of 3D scanning and traditional methods and found certain parameters of the foot were more reliably measured than others [23–25, 27] (Additional file 5). Across three studies, intra-rater reliability for foot length, forefoot width, and heel width all rated moderate to excellent for 3D scanning (ICC: 0.99 [24], 0.75–0.96 [23], and 0.87–0.96 [27]) and good to excellent for plaster casting (ICC: 0.91–0.99 [24], 0.91–0.95 [23], and 0.78–0.92 [27]) while foam impression box ranged from good to excellent (ICC: 0.93–0.95 [23] and 0.78–0.92 [27]). Two studies found clinical experience influenced intra-rater reliability, with more experienced clinicians in 3D scanning and traditional methods resulting in higher ICC values [24, 27]. When 3D scanning, the medial arch height intra-rater reliability was variable, considered excellent with ICCs of 0.96–0.97 [24], poor to moderate with ICCs of 0.43–0.70 [23] and 0.25–0.64 [27]. For traditional methods, medial arch height measured through casting ranged from moderate to good (ICC: 0.65–0.87 [24], 0.67 [23], 0.70–0.73 [27]) and foam impression box was scored poor (ICC: 0.49 [23] and 0.25–0.30 [27]). Intra-rater reliability for rearfoot/forefoot angle (RF/FF angle) was found to be good to excellent for 3D scanning (ICC: 0.75–0.96 [27]; 0.81–0.82 [24]; 0.65–0.79 [23]), ranged from poor to good when casting (ICC: 0.57–0.61 [27]; 0.36–0.49 [24]; 0.59–0.83 [23]), and was rated good for foam impression box (ICC: 0.79) [23].

The fourth study reported the intra-rater reliability between four methods of capturing the plantar surface of the foot parameters (3D scanning, digital calliper, digital footprint and ink footprint) in the following parameters foot length, forefoot width, rearfoot width [25]. The study found that 3D scanning had excellent reliability (ICC: 0.95–0.98), while reliability of digital callipers ranged from good to excellent (ICC: 0.74–0.98), digital footprint was excellent (ICC: 0.94–0.98) and ink foot print ranged from moderate to excellent (ICC: 0.59–0.98) [25].

## Discussion

In this systematic review of the literature, we identified six articles that compared 3D scanning with traditional methods of capturing morphology of the foot and/or ankle. There was moderate level evidence that 3D scanning requires less time to capture the foot and ankle when compared to traditional methods, especially for clinicians experienced in 3D scanning [26, 28]. The accuracy of 3D scanning for FOs was generally comparable with plaster casts, digital footprints and ink footprints to capture foot morphology [23, 25]. Head-to-head, 3D scanning was generally comparable in inter-rater reliability with plaster casting and foam impression as several foot parameters achieved excellent ICCs [23, 27]. The intra-rater reliability of 3D scanning and traditional methods was comparable with ICCs ranging between poor and excellent, however some aspects of the foot were more reliably captured than others. However, none of the included studies investigated accuracy or reliability of 3D scanning for AFOs which requires capturing lower leg, ankle as well as foot morphology. According to GRADE and COSMIN quality assessments, no study rated higher than moderate (Table 2) or adequate at best (Additional file 3) with most studies were assessed as inadequate or doubtful. Thus, there is insufficient evidence to support a clinical recommendation for the use of 3D scanning to capture the morphology of the foot and ankle (Table 5).

**Table 6:** Time required to cast or 3D scan the foot and/or ankle

	**Cast time (AFO)**		**3D Scan time (AFO)**		***p***-***value***
	All user	Experienced user only	All user	Experienced user only	
Roberts A, et al. 2016 [26]	12.2 (SD 4.9) min		11.1 (SD 9.5) min		*P* = 0.056
		13.1 (SD 4.4) min		8.9 (SD 2.9) min	*P* < 0.001
	**Cast time (FO)**		**3D Scan time (FO)**		***p***-***value***
	Student	Clinician	Student	Clinician	
Payne, 2007 [28]	16 min	11 min		2 min	

Two studies identified in this review reported the time required to 3D scan the foot and ankle, which is important when considering integration of digital processes into clinical workflows. Focusing on the scanning process, a study by Dessery and Pallari found that a low-cost 3D scanner (£289) was faster than a high-cost handheld scanner (£13,700) when capturing the knee for fabricating knee orthoses [30]. However, the Dessery and Pallari study did not compare the time taken for 3D scanning with a traditional method. When considering the entire orthotic device production workflow, one of the articles in our review, reported that 3D scanning in combination with CAM produced comparable devices as traditional methods in similar time frames [26]. More recent studies have supported these findings suggesting that additive manufacturing methods are as effective as traditional methods for producing AFOs when considering time, cost and device performance [5, 31, 32]. While some evidence suggests 3D scanning may be faster than traditional methods of morphology capture, it is crucial that this does not sacrifice accuracy or reliability.

With regard to reliability of capturing foot parameters, our review found that some foot parameters, such as length and forefoot width, were reliably captured regardless of the method used. Foot arch height was the least reliable foot parameter captured by any method. This could be due to the flexibility of the arch, which makes it difficult to hold in a consistent position when casting or scanning. Laughton et al., found that foot measurements were significantly influenced by the method used to obtain foot shape [23]. Similarly, Guldemond et al., compared FOs produced by plaster cast to foam impression box and observed that each method resulted in different pressure pattern and contact area [33]. Variability within capture position is significant as comfort, fitness, kinematic and kinetic function of the resulting orthotic device may be affected [34]. Too much variability in the 3D scanning process makes the correction of the deformity completely dependent on the rectification process, with several studies highlighting that this is a pinch point in the clinical workflow [26, 35, 36].

The experience of the clinician affected the reliability of the 3D scan in three of the included studies [24, 26, 27], and was related to reducing 3D scanning time and variability in all foot parameters. Therefore, user training and support is critical for successful implementation of 3D scanning processes in a clinical environment [37]. Roberts et al., found that clinicians inexperienced in 3D scanning had the most AFO fitting problems [26]. This is supported by Wong et al., who reported that clinicians who are familiar with CAD/CAM systems were faster and less likely to have fitting issues [35]. However, the learning curve for an orthotist to reach competence with a CAD/CAM system could be up to 4 years [36]. Despite improvements in reliability and speed with experienced users, it is unclear how much experience in 3D scanning is needed to produce quality scans and how quickly this is gained. Thus, continuous assessment of scanning accuracy and reliability as well as the regular evaluation of newly available technologies are recommended.

Our review only included studies that involved the foot and ankle. Inclusion of additional regions of the body may have resulted in higher article return in our search, however these were not relevant to our research question. We also did not assess the final orthotic device that was produced by either 3D scanning or traditional methods. However, other studies assessing AFOs [38], FOs [39, 40] and scoliosis braces [35, 36, 41, 42] found comparability between 3D scanning with those manufactured traditionally in terms of fitting and device performance. Most of the studies included in this review focused on the production of FOs for healthy adult participants, which does not account for deformities (such as the need to apply corrections during scanning). Only one study in our review investigated 3D scanning in children that were prescribed AFOs. The use of 3D scanning in children can be more difficult to manage considering adherence to instructions. Thus, there is a need for more high-quality studies that examine 3D scanning for AFOs for both paediatric and adult patients with pathology. In addition, there is a growing market for commercially available 3D scanners. Our review included laser 3D scanners, however there are many more models available. Therefore, as technology improves a continual assessment of the optimum 3D scanning method and technique in terms of speed, accuracy and reliability during scanning needs to be investigated.

## Conclusion

Studies comparing the speed, accuracy and reliability of 3D scanning with traditional methods of capturing the morphology of the foot and/or ankle are small and of generally low quality. With this limited evidence, 3D scanning was found to be faster than plaster casting for capturing the foot and ankle, especially for those experienced in 3D scanning. In the context of FOs, 3D scanning the foot is comparable in accuracy and reliability with traditional methods. However, this review found no evidence for the reliability or accuracy of scanning the foot and ankle for the fabrication of AFOs, an area ripe for future research.

## Supplementary Information

**Additional file 1:.** Medline keyword list.

**Additional file 2:.** Variations in data provided by each study.

**Additional file 3:.** Assessment of the methodological quality for each study following the COSMIN checklist.

**Additional file 4:.** Inter-rater reliability of methods of capturing foot morphology presented as ICCs.

**Additional file 5:.** Intra-rater reliability of methods of capturing foot morphology presented as ICCs.

## Data Availability

All data generated or analysed during this study are included in this published article [and its supplementary information files].

## Abbreviations

3D: Three Dimensional
AFO: Ankle Foot Orthosis
CAD: Computer-Aided Design
CAM: Computer-Aided Manufacturing
COSMIN: Consensus-Based Standards for the Selection of Health Measurement Instruments
FO: Foot orthosis
GRADE: Grading of Recommendations, Assessment, Development and Evaluations
ICC: Intraclass Correlation Coefficient
PRISMA: Preferred Reporting Items for Systematic Reviews and Meta-Analyses
