# Supplementary material for: Comparison of 3D scanning versus traditional methods of capturing foot and ankle morphology for the fabrication of orthoses: a systematic review
Source: J Foot Ankle Res. 2021 Jan 7;14:2. doi: 10.1186/s13047-020-00442-8 (PMC7792297; doi:10.1186/s13047-020-00442-8)
Supplement: Supplementary file 1 — Additional file 1:. Medline keyword list. [file 13047_2020_442_MOESM1_ESM.docx]

**Additional file 1:** Medline keyword list

| Ovid MEDLINE(R) ALL <1946 to March 06, 2020> | | |
| --- | --- | --- |
| # | Search Statement | Results |
| 1 | 3D scan*.mp. | 1127 |
| 2 | Photogrammetry/ or Photogrammetry scan*.mp. | 2449 |
| 3 | Computer-Aided Design/ or optical scan*.mp. | 16064 |
| 4 | Laser scan*.mp. | 21444 |
| 5 | Structured Light scan*.mp. | 85 |
| 6 | (3D Digitalization or 3D digitalisation).mp. | 4 |
| 7 | Three-dimensional construction.mp. | 46 |
| 8 | Three dimensional scan*.mp. | 347 |
| 9 | 3D surface scan*.mp. | 97 |
| 10 | 3d laser scan*.mp. | 312 |
| 11 | 3d Structured Light scan*.mp. | 14 |
| 12 | scan*.mp. | 631490 |
| 13 | 1 or 2 or 3 or 4 or 5 or 6 or 7 or 8 or 9 or 10 or 11 or 12 | 646904 |
| 14 | Ankle Joint/ or Ankle/ or ankle.mp. | 64727 |
| 15 | Foot/ or foot.mp. or Foot Orthoses/ or Foot Deformities/ or Foot Joints/ | 123789 |
| 16 | Shoes/ or Flatfoot/ or Foot morphology.mp. | 8422 |
| 17 | (ankle and foot).mp. | 18677 |
| 18 | ankle morphology.mp. | 19 |
| 19 | foot parameter*.mp. | 23 |
| 20 | ankle parameter*.mp. | 9 |
| 21 | 14 or 15 or 16 or 17 or 18 or 19 or 20 | 173112 |
| 22 | Plaster cast*.mp. | 2313 |
| 23 | Foot/ or Shoes/ or Computer-Aided Design/ or foam impression.mp. or Foot Orthoses/ or Orthotic Devices/ | 52442 |
| 24 | polyurethane resin.mp. | 74 |
| 25 | (fiber* cast* or fibre* cast*).mp. | 148 |
| 26 | (orthopedic cast* or orthopaedic cast*).mp. | 32 |
| 27 | cast*.mp. | 125768 |
| 28 | (Plaster Mold* or plaster Mould).mp. | 52 |
| 29 | (mold* or mould).mp. | 29239 |
| 30 | Hand cast*.mp. | 40 |
| 31 | (cast* adj4 ankle).mp. | 148 |
| 32 | (cast* adj4 foot).mp. | 151 |
| 33 | fiberglass cast*.mp. | 111 |
| 34 | Ankle Fractures/ or cast* socks.mp. | 1412 |
| 35 | negative impression.mp. | 132 |
| 36 | positive impression.mp. | 171 |
| 37 | foot Trace*.mp. | 2 |
| 38 | Foot Deformities, Acquired/ or Flatfoot/ or foot print.mp. | 4614 |
| 39 | 22 or 23 or 24 or 25 or 26 or 27 or 28 or 29 or 30 or 31 or 32 or 33 or 34 or 35 or 36 or 37 or 38 | 208947 |
| 40 | 13 and 21 and 39 | 1043 |
| 41 | footprint.mp. | 9681 |
| 42 | 39 or 41 | 218280 |
| 43 | 13 and 21 and 42 | 1057 |
