# Supplementary material for: Comparison of 3D scanning versus traditional methods of capturing foot and ankle morphology for the fabrication of orthoses: a systematic review
Source: J Foot Ankle Res. 2021 Jan 7;14:2. doi: 10.1186/s13047-020-00442-8 (PMC7792297; doi:10.1186/s13047-020-00442-8)
Supplement: Supplementary file 2 — Additional file 2:. Variations in data provided by each study. [file 13047_2020_442_MOESM2_ESM.docx]

**Additional file 2:** Variations in data provided by each study

| **Intervention/Methodology** | | | | | | | | | | **Outcomes** | | | | | | | | | |
| --- | --- | --- | --- | --- | --- | --- | --- | --- | --- | --- | --- | --- | --- | --- | --- | --- | --- | --- | --- |
| Reference | Device | | Participants | # of raters | | Scanner | | Situation | | Parameters | | ICCs | | | | | | Time | |
|  | AFO | FO | N | One | Two | Laser | Structure light | Partial weight bearing | No weight bearing | Foot (mean & SD) | Overall volume match | Intra caster | Inter caster | Intra-cad | Inter-cad | Reliability within-method | Validity: | Casting | Scanning |
| Carroll et al., 2011 (26) |  | x | 21 |  | x |  | x |  | x | x |  | x | x |  |  |  |  |  |  |
| Telfer et al., 2012 (28) |  | x | 22 |  | x | x |  | x |  |  | x | x | x | x | x |  |  |  |  |
| Laughton et al., 2002 (27) |  | x | 15 | x |  | x |  | x | x | x |  |  |  |  |  | x | x |  |  |
| Roberts et al., 2016 (25) | x |  | 153 | x |  | x |  | x | x |  |  |  |  |  |  |  |  | x | x |
| Payne, 2007 (30) |  | x | 1 |  | x |  |  |  | x |  |  |  |  |  |  |  |  | x | x |
| Lee, et al., 2014 (29) |  |  | 130 | x |  | x |  | x |  | x |  | x |  |  |  | x | x |  |  |
