# Supplementary material for: Comparison of 3D scanning versus traditional methods of capturing foot and ankle morphology for the fabrication of orthoses: a systematic review
Source: J Foot Ankle Res. 2021 Jan 7;14:2. doi: 10.1186/s13047-020-00442-8 (PMC7792297; doi:10.1186/s13047-020-00442-8)
Supplement: Supplementary file 3 — Additional file 3:. Assessment of the methodological quality for each study following the COSMIN checklist. [file 13047_2020_442_MOESM3_ESM.docx]

**Additional file 3:** Assessment of the methodological quality for each study following the COSMIN checklist**.**

| **Method** | **Reference** | **Measurement property: methodological quality per study** | | |
| --- | --- | --- | --- | --- |
|  |  | **Reliability** | **Measurement error** | **Criterion validation** |
| **Casting** | | | | |
| NWB | (26) | Inadequate (-)^*^ | Inadequate (+)^†^ |  |
| NWB | (27) | Inadequate (-)^*^ |  | Doubtful (+)^§^ |
| NWB | (28) | Inadequate (-)^*^ | Inadequate (?)^‡^ |  |
| **Foam impression** | | | | |
| PWB | (27) | Inadequate (-)^*^ |  | Doubtful (-)^§^ |
| PWB | (28) | Inadequate (-)^*^ | Inadequate (?)^‡^ |  |
| FWB | (28) | Inadequate (-)^*^ | Inadequate (?)^‡^ |  |
| **Ink** **footprint** | | | | |
| 50%WB | (29) | Adequate (-)^\|\|^ | Doubtful (?)^¶^ | Inadequate (?)^#^ |
| **Digital footprint** | | | | |
| 50%WB | (29) | Adequate (+)^\|\|^ | Doubtful (?)^¶^ | Inadequate (?)^#^ |
| **3D Scanning** | | | | |
| NWB | (26) | Inadequate (+)^*^ | Inadequate (+)^†^ |  |
| NWB | (27) | Inadequate (-)^*^ |  |  |
| PWB | (28) | Inadequate (-)^*^ | Inadequate (?)^‡^ |  |
| PWB | (27) | Inadequate (+)^*^ |  | Doubtful (-)^§^ |
| PWB | (28) | Inadequate (-)^*^ | Inadequate (?)^‡^ |  |
| PWB (corrected position) | (28) | Inadequate (-)^*^ | Inadequate (?)^‡^ |  |
| 50%WB | (29) | Adequate (+)^\|\|^ | Doubtful (?)^¶^ | Inadequate (?)^#^ |
| Abbreviations: NWB: non-weight bearing, PWB: partial-weight bearing, FWB: Full-weight bearing, 50%WB: 50%. weight bearing  Note: Rating based on COSMIN 4-point scale, but not inclusive of all COSMIN measurement property items  Methodological Quality: Very good, Adequate, Doubtful, Inadequate;  Outcome Quality: + = positive, - = negative, ? = indeterminate. | | | | |
| ^*^ Inadequate number of patients/volunteers (N < 30) for reliability.  ^†^ Inadequate number of patients/volunteers (N< 30) for measurement error. | | | | |
| ^‡^ Inadequate number of patients/volunteers (N< 30) for measurement error and no SEM defined.  ^§^ N < 30 patients/volunteers in biggest group for criterion validity.  ^\|\|^ ICCs calculated but no description of the model or formula.  ^¶^ No SEM defined. Poor methodological design for precision evaluation.  ^#^ No correlation or ICC was calculated. | | | | |
