# Supplementary material for: Comparison of 3D scanning versus traditional methods of capturing foot and ankle morphology for the fabrication of orthoses: a systematic review
Source: J Foot Ankle Res. 2021 Jan 7;14:2. doi: 10.1186/s13047-020-00442-8 (PMC7792297; doi:10.1186/s13047-020-00442-8)
Supplement: Supplementary file 4 — Additional file 4:. Inter-rater reliability of methods of capturing foot morphology presented as ICCs. [file 13047_2020_442_MOESM4_ESM.docx]

**Additional file 4:** Inter-rater reliability of methods of capturing foot morphology presented as ICCs

| **Parameters** | | **Foot length** | **Forefoot width** | **Rear foot width** | **Medial arch height** | **Rearfoot/**  **forefoot angle** |
| --- | --- | --- | --- | --- | --- | --- |
| Carroll, et al 2011 (27) | 3D scanning  (NWB) | 0.99 | 0.99 | 0.99 | 0.96 | 0.81 |
|  | Plaster cast (NWB) | 0.99 | 0.94 | 0.92 | 0. 87 | 0. 57 |
| Telfer, et al 2012 (29) | 3D scanning (relaxed standing 50% WB) | 0.92 | 0.81 | 0.93 | 0.73 | 0.83 |
|  | 3D scanning (corrected standing 50% WB) | 0.94 | 0.77 | 0.93 | 0.35 | 0.77 |
|  | 3D scanning (corrected sitting PWB) | 0.92 | 0.81 | 0.91 | 0.18 | 0.75 |
|  | Plaster cast (NWB) | 0.88 | 0.89 | 0.86 | 0.64 | 0.65 |
|  | Foam impression (sitting PWB) | 0.93 | 0.90 | 0.91 | 0.41 | - |
|  | Foam impression (walking FWB) | 0.88 | 0.89 | 0.88 | 0.48 | - |
| Abbreviations: NWB: non-weight bearing, PWB: partial-weight bearing, FWB: Full-weight bearing, 50%WB: 50% weight bearing | | | | | | |
