# Supplementary material for: Comparison of 3D scanning versus traditional methods of capturing foot and ankle morphology for the fabrication of orthoses: a systematic review
Source: J Foot Ankle Res. 2021 Jan 7;14:2. doi: 10.1186/s13047-020-00442-8 (PMC7792297; doi:10.1186/s13047-020-00442-8)
Supplement: Supplementary file 5 — Additional file 5:. Intra-rater reliability of methods of capturing foot morphology presented as ICCs. [file 13047_2020_442_MOESM5_ESM.docx]

**Additional file 5:** Intra-rater reliability of methods of capturing foot morphology presented as ICCs

| **Parameters** | | | **Foot length** | **Forefoot width** | **Rear foot width** | **Medial arch height** | **Rearfoot/**  **forefoot angle** |
| --- | --- | --- | --- | --- | --- | --- | --- |
| Carroll, et al 2011 (27) | 3D scanning (NWB) | R1 | 0.99 | 0.99 | 0.99 | 0.97 | 0.82 |
|  |  | R2 | 0.99 | 0.99 | 0.99 | 0.96 | 0.81 |
|  | Plaster cast (NWB) | R1 | 0.99 | 0.94 | 0.91 | 0.87 | 0.49 |
|  |  | R2 | 0.99 | 0.92 | 0.96 | 0.65 | 0.36 |
| Laughton, et al 2002 (28) | 3D scanning (PWB) | | - | 0.96 | 0.93 | 0.70 | 0.79 |
|  | 3D scanning (NWB) | | - | 0.75 | 0.78 | 0.43 | 0.65 |
|  | Plaster cast (NWB) | | - | 0.92 | 0.91 | 0.67 | 0.83 |
|  | Foam impression (PWB) | | - | 0.95 | 0.93 | 0.70 | 0.79 |
| Telfer, et al 2012 (29) | 3D scanning (relaxed standing 50% WB) | R1 | 0.94 | 0.87 | 0.96 | 0.64 | 0.96 |
|  |  | R2 | 0.92 | 0.87 | 0.95 | 0.50 | 0.93 |
|  | 3D scanning (corrected standing 50%WB) | R1 | 0.90 | 0.89 | 0.93 | 0.47 | 0.75 |
|  |  | R2 | 0.94 | 0.94 | 0.95 | 0.50 | 0.85 |
|  | 3D scanning (corrected sitting PWB) | R1 | 0.90 | 0.87 | 0.95 | 0.43 | 0.86 |
|  |  | R2 | 0.95 | 0.94 | 0.95 | 0.25 | 0.75 |
|  | Plaster cast (NWB) (R1, R2) | R1 | 0.87 | 0.87 | 0.91 | 0.73 | - |
|  |  | R2 | 0.86 | 0.91 | 0.87 | 0.70 | - |
|  | Foam impression (sitting PWB) | R1 | 0.90 | 0.90 | 0.90, | 0.30 | - |
|  |  | R2 | 0.87 | 0.90 | 0.92 | 0.25 | - |
|  | Foam impression (walking FWB) | R1 | 0.92 | 0.88 | 0.87 | 0.65 | - |
|  |  | R2 | 0.78 | 0.88 | 0.87 | 0.26 | - |
| Lee, et al 2014 (30) | 3D scanning (50%WB) | | 0.98 | 0.95 | 0.96 | - | - |
|  | Digital calliper (50%WB) | | 0.98 | 0.89 | 0.87 | - | - |
|  | Digital footprint (50%WB) | | 0.97 | 0.98 | 0.94 | - | - |
|  | Ink footprint (50%WB) | | 0.91 | 0.59 | 0.78 | - | - |
| Abbreviations: NWB: non-weight bearing, PWB: partial-weight bearing, FWB: Full-weight bearing, 50%WB: 50% weight bearing,  R1 = rater 1 (experienced user), R2 = rater 2 (less experienced user) | | | | | | | |
